# “White, Tall, Top, Masculine, Muscular”: Narratives of Intracommunity Stigma in Young Sexual Minority Men’s Experience on Mobile Apps

**DOI:** 10.1007/s10508-021-02144-z

**Published:** 2021-11-24

**Authors:** Phillip L. Hammack, Brock Grecco, Bianca D. M. Wilson, Ilan H. Meyer

**Affiliations:** 1 Department of Psychology, University of California, Santa Cruz, 1156 High Street, Santa Cruz, CA 95054 USA; 2 Social Science Interdepartmental Program, University of California, Los Angeles, Los Angeles, CA USA; 3 Williams Institute, UCLA School of Law, Los Angeles, CA USA

**Keywords:** Gay men, Stigma, Community, Social media, Intersectionality, Sexual orientation

## Abstract

What forms of intracommunity stigma do young sexual minority men narrate as they participate in communities through mobile apps? In a content analysis of 32 interviews with a racially diverse sample of young sexual minority men (ages 19–25; 84.4% non-White) from four regions of the USA, a majority of men (62.5%) spontaneously discussed mobile apps (e.g., Grindr, Scruff) when asked about their experience of community more broadly. Men’s narratives revealed engagement with intracommunity stigma related to body size, race/ethnicity, gender expression, and sexual position (e.g., bottom). Stigma related to HIV status, substance use, and social class were not spontaneously narrated in response to questions about men’s experience in communities. Expressions of stigma were frequently experienced intersectionally, particularly regarding racialized stereotype expectations (e.g., “Asian men are twinks, effeminate”). We discuss the ways in which sexual minority men reproduce dominant ideologies related to racism, misogyny, and masculine body ideals as they engage with one another on mobile apps. To the extent that many young men rely on mobile apps for community connection, their experiences of community might serve to exacerbate, rather than ameliorate, the deleterious impact of stigma.

## Introduction

Sexual minority men in the USA identify with such labels as gay, bisexual, pansexual, and queer and are stigmatized by dominant social norms governing both sexuality and gender (e.g., Hunt et al., 2016). But as members of a society with a long history of racism, misogyny, classism, and other stigmatizing ideologies, sexual minority men also perpetuate stigma when they uncritically internalize such ideologies (Hammack, 2018). What happens when stigma is experienced within the sexual minority community itself—a space typically considered a safe respite from a stigmatizing society and a buffer of minority stress (Meyer, 2003)?

In this paper, we examined the ways in which young sexual minority men (ages 19–25) narrate intracommunity stigma in their accounts of community, utilizing qualitative methods to allow men the openness to define “community” as they experienced it. While we assumed that “community” might evoke a sense of shared identity such as sexual or racial identity, we did not assume a uniform experience of belongingness. Hence we sought to directly interrogate the notion of a singular view of community for sexual minority men by foregrounding an intersectional lens which emphasizes relative privilege and power based on the particular configuration of one’s social identities (e.g., Bowleg, 2013).

We focused on the experience of young sexual minority men following a life course theoretical approach (e.g., Hammack et al., 2018a) which highlights the intersection of social time (i.e., membership in a generation-cohort) with developmental time (i.e., identity development in emerging adulthood). The men in our study were born between 1990 and 1997—members of the tail end of the “Millennial” generation (Dimock, 2019) and the early end of the “equality” generation of sexual minority men (Hammack et al., 2018a). They experienced childhood and adolescence at a time of growing recognition of the legitimacy of sexual diversity (e.g., marriage equality), and they were experiencing emerging adulthood in the 2010s at a time of new methods for social connection (i.e., mobile apps) and heightened awareness of issues related to intersectionality within sexual minority communities. Documenting the experience of community and stigma among sexual minority men of this generation constitutes a valuable new contribution to the literature and enhances our understanding of the social context of intracommunity stigma.

Methods for finding and connecting with other sexual minority men have changed drastically in the twenty-first century. The Internet and location-based social apps have revolutionized the ways in which young sexual minority men interact (Blackwell et al., 2015; Cassidy, 2018; Grosskopf et al., 2014; Gudelunas, 2012; Macapagal et al., 2020; Wu & Ward, 2018). These apps, which include among the most popular Grindr and Scruff, can be installed on one’s smartphone and then provide a visual grid of nearby individuals available for dating, sex, or social connection. These apps are widely used and have thus become vital tools for young people’s introduction to and integration into sexual minority communities (Baams et al., 2011; Miller, 2015; Renninger, 2019; Zervoulis et al., 2020). Given this context, we sought to understand *where* young men locate community (geographically, virtually, digitally) and explore the role of mobile apps in their conception of community and their experience of intracommunity stigma. Recognizing the competitive dynamics related to status that can occur within communities of sexual minority men (Pachankis et al., 2020), we focused on how intracommunity stigma is experienced as men engage with one another using mobile apps.

Recent research on sexual minority men’s use of mobile apps suggests complexity in the role of the apps in experiences of community. In a survey study of 191 men who have sex with men (MSM) in the UK, higher users of the apps reported a lower sense of community (Zervoulis et al., 2020). Respondents indicated that their primary motivation to use the apps was for sex or dating, with less than 5% of respondents indicating that their motivation was to connect to the gay community. In a qualitative study of LGBTQ + youth of diverse gender identities conducted in Australia, participants shared narratives of building friendships through experience on apps (Byron et al., 2021) and narratives of the way in which self-presentation on apps contributed to a greater ethical experience of “queer community” (Pym et al., 2021).

The purpose of our study was to examine narratives of intracommunity stigma in sexual minority men’s experience of community on mobile apps through an intersectional lens. Our intersectional framework considered the way in which men engaged with cultural narratives of race (e.g., White supremacy) and gender (e.g., conformity to heteronormative masculinity) which privileged proximity to power and thus denigrated diversity, creating negative psychological experiences for men. We focused on six forms of intracommunity stigma previously identified in the literature: body stigma, race/ethnicity stigma, gender expression/sexual position stigma, substance use stigma, social class stigma, and HIV stigma. We review research on each of these forms of stigma.

### Body Stigma

Like members of other sexual communities, sexual minority men encounter idealized notions of the body in response to which they view themselves as “attractive” or “unattractive” and potentially encounter stigma. For example, research suggests that sexual minority men experience pressure to conform to a lean and muscular physical ideal (e.g., Atkins, 2012; Calzo et al., 2013). Messages related to normative body ideals have been widely circulated on mobile apps since their inception, best embodied in the phrase “no fats, no fems” (Chow, 2021; see also Miller, 2018). The extent to which men publicly discuss “working out” on the apps also contributes to the promotion of a particular body ideal for sexual minority men (Chow, 2021; Miller, 2018).

Research has begun to document the way in which sexual minority men encounter idealized notions of the body on mobile apps, and theories have emerged to link objectification, social media use, and body image (Filice et al., 2020). Body type is key to the self-presentation practice for users of mobile apps like Grindr (Conner, 2019), with men asked to identify themselves with “tribes” that often index a specific body type (e.g., “bear,” “clean-cut,” “rugged,” “twink”). This process of self-categorization influences performances of gender that privilege certain body types over others (Conner, 2019). Among a national sample of 230 sexual minority men in the USA, the number of apps used was associated with body objectification and lower self-esteem (Breslow et al., 2020). In a qualitative study of 30 young sexual minority men in the USA, participants described the ideal male body as muscular, thin, and light-skin toned (Tran et al., 2020). They further described their experiences with discrimination on the apps and deviations from the normative ideal, with some noting the existence of body alternatives in the “bear” scene which privileges larger body sizes (Tran et al., 2020; see also Hennen, 2005; Manley et al., 2007). In a qualitative study of 13 Canadian users of Grindr, men’s narratives revealed the experience of weight stigma, sexual objectification, and social comparison related to the body (Filice et al., 2019). Of the 13 interviewees, nine indicated that the use of Grindr negatively impacted their body satisfaction.

### Race and Ethnicity Stigma

Sexual racism privileging White men and White bodies has a long history and remains common in communities of White sexual minority men (Han & Choi, 2018). Sexual minority men across racial identities experience racially stigmatizing behavior online (Callander et al., 2015). Explicit racism has been documented in user profiles on mobile apps such as Grindr, often taking the form of expressions of “preference” for partners of particular races (Conner, 2019).

Research has begun to document the experience of race/ethnicity stigma on mobile apps among sexual minority men of color. In a study of young Black sexual minority men in the USA, app users reported higher levels of racial discrimination than non-users (Rosengren et al., 2019). In their qualitative study of 30 young sexual minority men in the USA, Tran et al. (2020) found that men narrated common experiences of racism (e.g., encountering those seeking only White partners) and colorism (e.g., encountering those expressing a preference for lighter skin tones).

### Gender Expression and Sexual Position Stigma

Gender expression stigma occurs with the widespread stigmatization of femininity among sexual minority men, rooted in misogyny (Hale & Ojeda, 2018; Hammack, 2018; Wilson et al., 2010). Gender non-conforming sexual minority men experience discrimination not only from the broader society (Thoma et al., 2021), but also from other sexual minority men who express contempt and hostility toward effeminate men (Taywaditep, 2002). In addition to desiring masculinity in others, sexual minority men also strive to appear masculine to other sexual minority men (e.g., Sánchez & Vilain, 2012) and in their performances of gender in relationships (Lu et al., 2019). This preference within the community for masculine over feminine gender presentation can lead to both romantic and social rejection for sexual minority men who do not present as traditionally masculine (Skidmore et al., 2006).

Recent research has documented the way in which pressure to conform to masculine gender is embedded in men’s discourse and practice on mobile apps. In an online survey of sexual minority men primarily from the USA, Canada, and other Anglophone societies, researchers found that longer term usage of mobile apps was associated with a decline in self-perceived masculinity (Miller & Behm-Morawitz, 2020). They interpret this finding as indicative of the way in which rigid norms around masculinity are cultivated on the apps and lead many men to question whether they adhere to standards. Qualitative research of British men’s speech on mobile apps supports the notion that many men use discourse that conforms to traditional heteronormative masculinity or a “straight-acting” style (Sarson, 2020). In a survey of sexual minority men in the USA, researchers found a preference for simulated mobile-app profile photos of White and Latino men whose features were rated as most consistent with heteronormative masculinity (Cascalheira & Smith, 2020).

Sexual position (i.e., top/bottom) has been associated with masculinity, with those who assume the top (i.e., insertive) role in anal sex being considered more masculine (e.g., Ravenhill & de Visser, 2017). Closely related to the privileging of masculine gender expression and the stigmatization of feminine gender expression among sexual minority men is stigmatization of identifying as a bottom (e.g., Underwood, 2012). In a survey of 282 sexual minority men in the USA, researchers found that those who identified as bottoms sought partners who identified as tops and were perceived as gender-typical (i.e., conforming to normative masculinity) and sexually dominant (Moskowitz & Roloff, 2017). In a qualitative study of 17 sexual minority men in the UK, researchers found that men who subscribed to mandates of hegemonic masculinity were more likely to conflate sexual position and gender (Ravenhill & de Visser, 2018).

### Substance Use Stigma

Affiliation with sexual minority men’s communities has been associated with higher rates of substance use (Compton & Jones, 2021). The higher frequency of substance use in these communities is related to social norms and cultural settings in which men interact, which have historically included nightlife settings but now also constitute mobile apps (e.g., Gaspar et al., 2021; for review, see Boyle et al., 2020). While use of substances in some contexts (e.g., alcohol in bars) has long been commonly accepted as normative within sexual minority communities (Boyle et al., 2020), it has been stigmatized in other contexts (e.g., crystal methamphetamine and other substances in anonymous sex in homes; see Frederick & Perrone, 2014; Heritage & Baker, 2021). The latter context has become increasingly common since the advent of mobile apps and is commonly referred to as “chemsex” to signal the intentional use of recreational substances to enhance the sexual experience (e.g., Ahmed et al., 2016).

The experience of stigma for either using or not using substances is complex, with some men reporting pressure to use substances (e.g., Tan et al., 2018) while others reporting stigma for use of particular substances such as crystal methamphetamine (e.g., Treloar et al., 2021). In a qualitative study of 30 sexual minority men (ages 18–39) in Singapore, men reported fear of rejection from sexual partners on mobile apps as a motivation to engage in chemsex (Tan et al., 2018). In a qualitative study of 20 sexual minority men (ages 21–66) in Vancouver, Canada, researchers found that men reported substance use as an important social activity for inclusion in the community (Hawkins et al., 2019). However, they found that men distinguished between different substances, stigmatizing use of drugs such as heroin and methamphetamine (Hawkins et al., 2019). Among a sample of informants who used crystal methamphetamine in Australia, men reported that substance use was beneficial to reduce internalized stigma associated with same-sex desire, but they reported stigma for methamphetamine use by the larger community and its resource-based organizations (Treloar et al., 2021).

### Social Class Stigma

Lower perceived social class is a source of stigma within communities of sexual minority men (Coston & Kimmel, 2012). A tension centered on popular community representation has led to marked stratification, particularly between middle-class and working-class sexual minority men (Gluckman & Reed, 2012; Heaphy, 2011). Despite evidence to the contrary (Badgett et al., 2019), sexual minority men have been portrayed in popular media as largely upper middle-class. Those men who happen to fit this stereotype are privileged in defining what it means to be a gay, bisexual, pansexual, or queer man (Barrett & Pollack, 2005; Valocchi, 1999). In contrast, working-class sexual minority men can experience greater psychological distress (Gamarel et al., 2012) or feelings of exclusion that can lead to less involvement with sexual minority communities (Barrett & Pollack, 2005; Connell et al., 1991).

Though research on class dynamics among sexual minority men is limited, existing research reveals conflict and division. In an interview study of 39 working-class sexual minority men in the USA, men described conflict between working-class and middle-class men in terms of a clash of values and mindsets (Appleby, 2001). Interviewees expressed resentment toward middle-class men, who they saw as having different norms around family and community and who they experienced as often objectifying them. In an interview study of 48 sexual minority men in the UK, middle-class men described working-class culture as inherently homophobic and intolerant (Heaphy, 2011).

The role of social class in men’s experience of mobile apps has rarely been studied. In a qualitative study of sexual minority men in the UK, income emerged as a factor which influenced men’s use of apps, namely limiting accessibility for men with lower incomes (Davis et al., 2016). No research to our knowledge has directly examined the experience of social class stigma on mobile apps used primarily by sexual minority men.

### HIV Stigma

There is a long history of stigma based on HIV-positive status within communities of sexual minority men (for review, see Smit et al., 2012). When mobile apps such as Grindr first emerged in 2009, “serosorting” was a common HIV prevention strategy in which individuals chose their sexual partners based on shared HIV status (Parsons et al., 2005). This approach to safer sex highlighted and likely further stigmatized HIV-positive men (e.g., Courtenay-Quirk et al., 2006).

With the advent of pre-exposure prophylaxis (PrEP) for HIV, as well as greater awareness about the low transmission likelihood of those with undetectable viral loads, there may be changes and more nuances related to HIV stigma. For example, there is stigma about the use of PrEP itself, with many men invoking discourses of sexual shame with the language of the “PrEP whore” (Dubov et al., 2018; Hammack et al., 2019). On the other hand, fear of contracting HIV has been reduced with these innovations in treatment and prevention (Hammack et al., 2019). Stigma about HIV status or PrEP use may be driven by local community norms. Sexual minority men who reside in an urban center, where PrEP use is more common, are less stigmatized (Hammack et al., 2018b).

The experience of HIV status stigma on mobile apps has not been widely investigated. In a qualitative study of sexual minority men in the USA, men used words such as “clean” to describe their HIV-negative status on Grindr (Conner, 2019). HIV-positive men in the study narrated the psychological challenges of encountering such language in their use of the apps. Discourses related to PrEP contained references to sexual shame and promiscuity (Conner, 2019).

### The Current Study

Grounded in life course and intersectionality frameworks that center the location of individuals in social and historical time, our study examined narratives of intracommunity stigma among a diverse sample of sexual minority men in the USA. We focused on young men of a particular generation (born between 1990 and 1997; ages 19–25 at the time of study) who experienced the emergence of mobile apps as new primary sites of community connection at a critical period of development: early emerging adulthood. Our life course and intersectionality framework highlights the way in which men who share a generational location but diverge on other social identities of varying proximity to traditional axes of power (e.g., race, class, gender conformity) experience stigma. This theoretical application is novel and stands to contribute to our understanding of the meaning and experience of stigma in context.

Informed by an interpretive, contextualist epistemology (Josselson & Hammack, 2021; Madill et al., 2000), our goal was to understand the experience and meaning of stigma experienced within communities of sexual minority men. We assumed that these narratives reflected individual interpretations of social experience (“narrative truth”) and that these narratives provided access to meaning making processes for our interviewees (see Bruner, 1990; Spence, 1984). Following an interpretive approach to qualitative data analysis, fidelity was achieved through multiple readings of the data and the establishment of consensus among members of the research team (see Levitt et al., 2017, 2018).

## Method

### Participants and Procedure

This paper used data collected as part of the qualitative component of the Generations Study, a mixed-methods study examining identity, stress, and health in three generations of sexual minorities in the USA (see Frost et al., 2018, 2020). A quota sampling approach was used to achieve roughly equal representation of participants across age cohort, gender, race/ethnicity, and geographic region. Many qualitative studies of sexual minority people recruit from within a single geographic region, which limits the ability to capture a diversity of lived experience. To address this concern, we recruited participants from four distinct regions of the USA: Austin, Texas; New York City; San Francisco Bay Area; and Tucson, Arizona. Each site had a catchment area of 80 miles, which allowed us to recruit interviewees from more rural areas as well. Researchers closely monitored interested interviewees to achieve the roughly equal representation across indicators listed above (for more information, see Frost et al., 2018).

We used a venue-based recruitment strategy in which ethnographic methods were first used to identify key sites frequented by sexual minority people in the four regions. Venues included stores, cafes and restaurants, churches/temples, parks and other outdoor areas (street), bars and clubs. To minimize bias inherent to community samples, we avoided recruitment from venues that, by design, over-represent individuals with high levels of mental health problems and/or stressful life events (e.g., 12-step programs, HIV/AIDS service providers). To reach individuals who may not otherwise attend the physical venues, targeted study advertisements were placed in local social media. Trained research workers recruited individuals in these venues, providing study information including a study website and toll-free number where information and screening for eligibility was conducted. To avoid saturating the sample with attendees of any particular venue at a particular time, recruitment caps were used so that no venue was disproportionately represented in the sample. The final sample comprised 191 interviews.

Interviews were conducted in private locations such as university offices, public library rooms, or rented office space between April 2015 and April 2016. Since the focus of our analysis was on young sexual minority men, our dataset consisted of the 32 men who were part of the younger cohort (ages 19–25). The average duration of interviews was 2.5–3 h and contained a flexible protocol organized by seven sections: life story (including life-line drawing activity); social identities and communities (including social identity mapping activity); sex and sexual cultures; challenges, stress, and coping; reflections on social and historical events related to sexual and gender diversity issues; healthcare utilization; and reflections and goals. Interviewees received a $75 USD incentive for their participation. Interviews were recorded using a digital audio recorder and transcribed by a professional transcription service.

For the analysis reported here, we analyzed the transcripts of all interviews from sexual minority men within the younger cohort from the larger study. Demographic information for the sample is provided in Table 1. Men chose their own terms to describe race or ethnicity. We note that all names used in this report are pseudonyms created to match participants’ actual names in terms of race/ethnicity. For example, if a Latino interviewee used an Anglicized name, we selected an Anglicized pseudonym.

**Table 1:** Interview sample demographics (*N* = 32)

Characteristic	*N*	%
*Age (in years*)		
19	3	9.4
20	4	12.5
21	6	18.8
22	5	15.6
23	8	25.0
24	5	15.6
25	1	3.1
*Interview site*		
Austin, Texas area	9	28.1`
New York City area	8	25.0
San Francisco Bay area	8	25.0
Tucson, Arizona area	7	21.9
*Race/ethnicity*		
American Indian	3	9.4
Asian/Pacific Islander	5	15.6
Bi/multiracial	5	15.6
Black/African-American	6	18.8
Latino/Hispanic	8	25.0
White	5	15.6
*Sexual identity*		
Bisexual	4	12.5
Gay	26	81.3
Pansexual	1	3.1
Queer	1	3.1
*Education level*		
High school	7	21.9
Some college	15	46.9
Associate’s degree	1	3.1
Bachelor’s degree	7	21.9
Some postgraduate	2	6.3

### Measures

The sections of the interview we analyzed asked about men’s social identities and communities, as well as sex and sexual cultures (for detailed discussion of the interview protocol, see Frost et al., 2020). The social identities section typically occurred about 30 min into the interview. Interviewees completed an identity mapping activity in which they were asked to list “identities and roles that describe who you are.” They were primed that these identities might include “labels related to gender, race, sexuality, class, occupation.” The participants were then asked to provide narratives intended to capture intersectionality of experience across communities of particular social identities. For example, an interviewee who identified as queer, South Asian, and male would be asked, “What is your experience of being queer in the larger South Asian community?” and “What is your experience of being South Asian in the larger queer community?”.

The section on sex and sexual cultures typically immediately followed the social identities section. Interviewees were asked to discuss their experiences of sex, dating, and relationships, including sexual roles (e.g., top, bottom). Interviewers probed for details of their experience of sexual culture, including norms, practices, and standards of attraction.

We note that the protocol did not ask any specific questions regarding intracommunity stigma. Men’s narratives of stigma thus occurred spontaneously in their more general discussions of identities, communities, and sexual cultures.

### Data Analysis: Interview Coding

Data were analyzed using the Dedoose application for qualitative analysis. Our analysis of interview data blended inductive and deductive approaches. First, we conducted an inductive analysis of narratives of community to discern the primary contexts in which men experienced community (e.g., bars, gyms, online/apps). Second, we conducted a directed content analysis (Hsieh & Shannon, 2005) in which our initial coding was guided by the sources of intracommunity stigma identified in the literature and reviewed above. We present the results of both our inductive and deductive analyses to represent the narratives that emerged about the experience of intracommunity stigma among our sample. Our selection of excerpts was guided by a principle of representativeness within each content category.

During the coding process, the second author annotated all mentions of words, phrases, and ideas indicative of stigma (experiences of prejudices, discrimination, violence). At regular intervals, the second author shared the coded excerpts with the fourth author in order to ensure consistency in coding, address any disagreements, and foster a reflexive dialogue. The entire authorship team then constituted a larger interpretive community through which the data excerpts and analysis presented here achieved fidelity (see Levitt et al., 2017). For reflexivity, we note that the four authors embodied a diverse interpretive team with regard to gender, sexuality, and race (identifying with such identities as man, woman, gay, lesbian, queer, Black, and White).

## Results

### Centrality of Mobile Apps in Narratives of Community

A key finding from our inductive analysis was the centrality of mobile apps in men’s experience of community. When asked about their experience of community, the majority of interviewees (20 of 32; 62.5%) spontaneously invoked mobile apps such as Grindr, Scruff, and GROWLr. This finding suggested that these apps served as key sites of experiences of intracommunity stigma.

Mobile apps were spontaneously mentioned as a site where various stigmas were perpetuated and experienced. For example, when asked about being “African-American within the gay community,” Shawn, a 21-year-old, gay, Black interviewee from the San Francisco Bay area whose highest education was a high school degree responded by describing his negative experiences on apps: “Anytime I message somebody on GROWLr or Grindr or Scruff, I would never get a response back.” The implication of Shawn’s narrative is that a primary site of community is one in which he does not even feel seen or recognized.

Asked about being “Latino in the gay community,” another San Francisco Bay area interviewee, Salvador, a 20-year-old, gay, Latino man whose highest education was a bachelor’s degree also spontaneously discussed his experience with apps:

…I’m not getting a whole bunch of attention from Scruff and Grindr and GROWLr. It just doesn’t happen. . . .They’re on all of the apps that I’m on or sometimes they’re different, but there’s just so many men on Scruff, on Grindr, and all these apps that I’m on. They don’t even think about looking at me.

Similar to Shawn, Salvador narrated an experience of non-recognition on mobile apps—an experience of exclusion from what he perceives as the dominant source of community for sexual minority men.

When asked about his experience as a “male within the LGBT community,” Daniel, a 23-year-old, gay, Latino with some college education from the New York City area, highlighted the centrality of the apps:

It is difficult cause, like I’ve said, been trying to date with like a relationship, but it’s hard because one, I don’t…go to gay bars or anything. I more so do everything online. It’s annoying because online the majority of men on online gay sites or apps are looking for sex… It’s annoying because it’s half the messages I get on [Grindr] that it’s people wondering what’s my sexual preference, top/bottom, etcetera, or sending nude photos. It was just annoying cause it seems like half of all gay men just want sex.

Daniel’s narrative revealed the perceived limitations of the mobile apps as sources of potential romantic relationships, with a focus that he perceived as sexual.

These excerpts reveal the centrality of mobile apps in the spontaneous narration of community for young sexual minority men and are representative of the men in our study. This finding emerged inductively in our analysis of the narrative data. In the remainder of the Results section, we reveal findings from our directed content analysis of the identified forms of intracommunity stigma we expected to find: body stigma, race/ethnicity stigma, gender expression and sexual position stigma, substance use stigma, social class stigma, and HIV stigma.

### Body Stigma: “Just Don’t Be Too Heavily Fat Like Rasputia”

Experiences of and references to body stigma were prevalent across interviews. While discussing the role that his body plays in his attempts at intimacy, Luke, a 20-year-old, White, pansexual man with some college education from the Austin, Texas area, explained:

I have this insecurity that if I’m with someone intimately, and they see my body, that they won’t want to talk to me after that. I don’t really like the way my body is.

Luke’s narrative suggests internalization of a particular physical ideal—an ideal he sees as most desirable to other men.

Daniel echoed Luke’s sentiments when talking about losing weight:

…Physically, I was just, God, fat. I also had pretty long hair for a while cause I was donating it. And then glasses. I just was like totally what no one would [laughter] find attractive. My first relationships were for mostly just sex cause I felt like no one would want me as, like, a boyfriend. But then I lost the weight. I got some confidence back. I was able to find more guys who were attracted to me and more guys who would actually wanna go out on dates with me.

Daniel’s narrative demonstrated both the internalization of a particular body ideal within the community of sexual minority men—an ideal which he felt he did not initially embody—and that that ideal has implications for both how he sees himself and his prospects for intimacy. Having lost weight, Daniel now sees himself as desirable to other men.

Justin, a 20-year-old, gay, multiracial man with some college education from the New York City area, expressed his own preferences related to body size and said that there is “nothin’ wrong” with expressing such preferences.

A lotta people’s into what they into. With me? No. Just don’t be too heavily fat like Rasputia.

In his narrative, Justin perpetuated the types of stigmatizing attitudes about body size that other participants described as hurtful. For example, Salvador described feeling marginalized in the community because of his body size, saying “I am definitely a chub... not fat.”

I can’t identify community with the queer community because we’re just—as bigger people we’re just marginalized. We’re so marginalized. I am abnormal in that sense. I can’t identify with that. Even with the one community that I most identify with, I can’t identify with it completely.

Salvador’s narrative revealed how men’s experience of body stigma extends beyond their experience with possible sex partners to the larger community’s body standards and creates challenges for identification and a sense of inclusion.

Max, a 19-year-old, gay Asian man from the San Francisco Bay area whose highest education was a high school degree, narrated the pressure that he experienced to adhere to body stereotypes within the gay community.

Part of being gay that really affected me because—it was my body and my overweight. Being gay, the stereotype is buff and clean-looking and nice and everything. That did affect me a little where my self-esteem about my looks were going down. That’s why I started eating more because—yeah, that did affect my depression a little, where I wasn’t comfortable with my own looks and I felt that I was ugly… In the gay community, everyone else is better looking.

Max’s narrative revealed the way in which internalization of body stigma can result in mental health challenges, including depression.

Max went on to link body expectations related to his race to his body stigma:

As well as being gay and Asian there’s a physical stereotype as well. Gay Asians are mostly, you would say, twinks and slim, etcetera. So that also relates with me being fat. It’s hard being a fat, gay Asian for that part.

Max’s narrative further revealed the way in which intracommunity stigma is often experienced intersectionally, based on racialized stereotypes within the larger community of sexual minority men.

### Race/Ethnicity Stigma: “I Don’t Usually Go for Black Guys”

Racism was experienced by men of color as they described both rejection and fetishization on mobile apps. Of the 16 men of color who discussed the apps as sites of community, seven (43.8%) spontaneously narrated experiences of race stigma on the apps. Two of the five White men interviewed discussed race/ethnicity stigma on the apps as well, revealing that some White men have an awareness of racism perpetuated on the apps.

Men of color narrated frequent encounters with public declarations signaling a lack of interest in men of color or a desire in relation to a particular racial stereotype (e.g., “thug”). They also narrated exclusion from virtual community spaces implicitly or explicitly marked as “White.”

Joseph, a 21-year-old, gay, Black man with some college education from the Austin, Texas area, described both the racial stigmatization and fetishization that he experienced on the apps:

I feel like Blacks in the gay community aren’t really accepted as much as any other race. You see the whole thing like “no femme, Blacks, gays or Asians”—“[no] fats or Asians” and stuff like that. . . . I just feel like I’ve been fetishized for my skin, my stature, and for some reason people think I’m gonna have a huge dong.

Joseph’s narrative revealed his experience of sexual racism, characterized both by feelings of exclusion and fetishization on the apps.

Zach, a 21-year-old, gay, Black man from the Austin, Texas area whose highest education was a bachelor’s degree, echoed Joseph’s experience of being simultaneously sexually desired and rejected due to his race:

I don’t care if you won’t date Black guys. I mean, obviously people are attracted to what they’re attracted to. I just think that for people to overtly proclaim, “I would never date a Black guy.” I think it’s weird. There’s that team of people. Then you have the other, who’s just way too into Black guys. One guy asked me to have sex with him in a do-rag and Timberland boots, or whatever. They want you to be the stereotypical, I guess, thug. Then within that group…they don’t always want you to be a thug. I remember one guy said something to me to the effect of, “Oh, I’m so glad I met you cause I’m really into Black guys. You’re actually educated.” Things like that. I’m just, “...Okay. That’s nice? I guess? Thank you.” Sarcastically, of course… Either they’re like, “Ew, gross, Black!” or they’re like, “Mandingo!”

Juan, a 23-year-old, gay, Latino man with some postgraduate education from the Tucson, Arizona area, discussed how his ability to pass as White created a distance between himself and other gay Latinos:

I guess when I think about it, I could say that being perceived as White has helped me through a lotta things. I definitely feel a disconnect between myself and other gay Latinos…

Like other men of color, Juan also talked about expectations of a particular gender expression and appearance associated with being a gay Latino man:

I feel like the stereotypical gay Latino might be somebody who’s more flamboyant or who—or people perceive them to be more flamboyant or feminine. Probably of darker skin . . .

Some men described feeling excluded from communities they described as dominated by White gay men. For example, Adrian, a 23-year-old, gay, Latino man from the San Francisco Bay area whose highest education was a high school degree, talked about the Castro, a historically gay neighborhood, saying Black and Latino youth “are having a hard time accessing those communities”:

I am left to think that it’s because it’s catered to a certain demographic, right? I’m left to think that it’s all catered to the gay White male, and the other races or people don’t feel as comfortable.

Similarly, Micah, a 22-year-old, gay, Black man with some college education from the New York City area, described his experience of exclusion as a Black man within the bear community—a subcommunity that celebrates weight diversity and yet can reproduce race stigma in creating its own normative standard (i.e., White, large, hairy; see Hennen, 2005; Manley et al., 2007).

I’m on Tumblr. Every time I go and some of these blogs, I’m just looking at these pictures of men. I’m just like, “So there are no Black bears or any bears of color out there that you couldn’t put one on your blog.” These guys are cute and all, but none of these—like what? I think it’s really tough because you’re either typecast as a “thug” or someone who is uneducated, like, period. When I go to bear parties or when I’ve gone to the club…I’m like, “I don’t feel comfortable here. I don’t see anybody who looks like me.” I remember one time I had gone out, and some guy came up to me. He was like, “You’re not usually my type.” I’m like, “What’s that supposed to mean?” He was like, “I don’t usually go for Black guys.” I was like, “Yeah, please stop talking to me. I don’t want to—let’s not do this.”…I feel like really weird sometimes being a Black gay man. Around other people—basically White gay men—because it almost feels like they don’t see you for anything more than your skin color.

Micah’s narrative revealed a sense of exclusion from spaces dominated by gay White men. Such spaces represent sites within communities in which intracommunity stigma such as sexual racism is enacted.

Salvador’s narrative interchangeably talked about community and apps to highlight his experience with sexual racism.

*Salvador: *In the terms of…being a Salvadoran Latino within the queer community, I feel as if there’s a lot to be done and there’s a lot of racism within the queer community, regardless of how accepting we ask the rest of the world to be towards us and how liberal we tend to be… I just don’t know how many times on Grindr, on Scruff or on GROWLr—on all these gay hookup sites apps I’ve seen several people who either say, “Latino+++” or they’ll say “White only” or they’ll say “Asian+++” or “Asian only” or something like that.

*Interviewer:* What’s “ +  +  + ”?

*Salvador:* “ +  +  + ” means benefit. Like, “That’s what I like.” …It’s a plus.

Salvador’s narrative revealed the centrality of sexual racism in his experience of the larger community of sexual minority men. For Salvador and other men of color, engagement with the broader community of same-sex attracted men inherently requires a confrontation with racism.

### Gender Expression and Sexual Position Stigma: “I Have to Put on a Façade”

Expectations of masculine gender expression were common throughout men’s narratives. Bryan, a 22-year-old, gay Latino man with some college education from the Tucson, Arizona area, identified as masculine and explicitly denigrated feminine sexual minority men, socially and sexually, even at the expense of participating in in-person sexual minority communities.

There’s a lot of feminine guys, and because of that I avoid them and try to hang out with the more masculine guys. Maybe that cuts me off from the LGBT community because I feel like the ones who are in Pride or in GSA tend to be more on the feminine side. I don’t go to those Pride lines or anything. I feel like the clubs or music dancing, pop music, so I don’t really go to those. My masculinity I guess keeps me away from it and forces me to go on Grindr and the online dating sites to find guys who may be hiding or who also you wouldn’t know unless you are looking for them on an app or something. It makes it harder for me to meet guys in person because I don’t go to the places where the other gays would be.

Bryan’s narrative revealed a denigration of femininity and the perception of men who participate in much of the LGBT community as feminine in their gender expression. Subsequently, he saw his only source of community as Grindr and sought masculine-presenting men there.

Fernando, a 21-year-old, gay, Latino man with some college education from the Austin, Texas area, also narrated the privileging of masculine gender expression in the community on the apps:

…[A] lot of people dislike someone just because they’re feminine, so aggressively may I add. This is something I’m also working on. Because I might be coating it. It’s like, “Oh I don’t hate them because they’re feminine, I hate them because they’re annoying.” I find them annoying because they’re feminine. It’s also something I need to work on. I think it’s always safer to play masculine whenever I have the opportunity. Feminine is more of a luxury I guess if I had to put it.

Unlike Bryan, Fernando was critical of the denigration of femininity in men’s communities and expressed his own self-work on this issue. That is, Fernando recognized the denigration of feminine gender expression as a problem to be addressed, whereas Bryan uncritically adopted this ideology in his personal narrative. Fernando’s framing of feminine gender expression as a “luxury” is interesting and suggests that masculine presentation represents a possible defense against violence for some sexual minority men.

While gender expression and one’s preferred sexual position are not inherently related, the way many participants discussed these issues indicated they equated masculinity with being a top and femininity with being a bottom. Underlying the privileging of masculine gender expression and the “bottom shaming” described by men appeared to be a denigration of femininity.

Luke discussed how his feminine gender expression created challenges as he prefers to take a dominant role in sex.

A lot of the time, I have to put on a façade and not present as feminine, especially if I want someone to be attracted to me as the dominant partner. I used to have a picture where the way I was posing, you could see that my nails were painted. . . . I really liked the way my face looked in the pictures, but I was like, “Okay. Well, I’ll just get rid of that . . .” I won’t have any picture on my dating websites where my hair’s down. It makes me look more feminine.

Luke narrated that community norms associate feminine gender expression with more passive sexual roles. This led to a conflict between his presentation—required to project a desirable image—and being authentic.

Men discussed intentional masculine gender expression within sexual encounters. The narrative of Matt, a 24-year-old, queer, multiracial man from the Tucson, Arizona area whose highest education was a high school degree, illustrates.

I do notice definitely that when I’m having sex, I turn on the masculinity a lot more, just because I know that’s more likely to turn on the other person, just because—I don’t know—femininity doesn’t turn people on. …Yeah, cause I still have a lot of that femmephobia in my sex life.

Matt acknowledged feminine gender expression as a source of stigma—especially in sexual encounters—in communities of sexual minority men, and he explicitly named femmephobia as an internalized ideology.

Brandon, a 23-year-old, gay, White man from Tucson, Arizona whose highest education was a bachelor’s degree, explained the importance of sexual position identities to his partner.

I have to be careful not to use terms like mentioning about him bottoming because he’s very insecure about he doesn’t wanna be considered a bottom. I have to tread carefully in considering myself top. We both consider ourselves to some degree a vers, or versatile, leaning one way or the other slightly.

The sensitivity about identifying as a bottom that Brandon and other men discussed reveals the way in which this role—associated with femininity—is experienced as stigmatized within the community for some men.

### Substance Use, Social Class, and HIV Stigma

Our directed content analysis did not discover substantial content in any of the other domains of potential stigma identified by the literature, including substance use, social class, or HIV status. Further, no other themes relevant to stigma emerged.

## Discussion

Like all men in contemporary US society, young sexual minority men are socialized in a larger cultural context of racism (Roberts & Rizzo, 2021), misogyny (Hale & Ojeda, 2018), and pressures to conform to a particular body standard (Murnen & Karazsia, 2017). In the course of their development, they might either repudiate or reproduce these ideologies as they form their identities in and through community experience (Hammack, 2018). Following a life course and intersectional theoretical approach and an interpretive epistemology, we sought to understand the way in which men who experienced emerging adulthood with the rise of mobile apps as key sites of community connection engaged with these ideologies.

In this study, we examined narratives of intracommunity stigma in young sexual minority men’s experiences of community, the majority of whom spontaneously narrated as occurring on mobile apps. Although respondents were asked by interviewers about their experiences in the “community,” most understood that to relate to their experience with apps such as Grindr and Scruff. This finding is consistent with other research that has revealed the central role of mobile apps in the lives of young sexual minority men, particularly in formulating, finding, and understanding their place within sexual minority communities (e.g., Cassidy, 2018; Hughto et al., 2017; Jaspal, 2017; Macapagal et al., 2020; Renninger, 2019; Taylor et al., 2017).

In contrast to men of prior generations, whose social development was limited by physical boundaries of geography, men of younger generations such as the late Millennials in this study experienced the social world at an early age as online (e.g., Manago, 2015). Thus, it is not surprising that they turn to mobile apps in the course of their development to form social and sexual connections with other men. While there is the advantage of accessibility, particularly for sexual minority people in rural or more conservative geographic locations (e.g., Van De Wiele & Tong, 2014), the shift to online settings as a major source of community building creates challenges, given that most men report they are motivated mainly to seek sex on the apps (Zervoulis et al., 2020).

Our analysis revealed that apps represented a context in which men experienced intracommunity stigma and were thus associated with negative experiences. This finding supports research linking use of social networking apps to lower self-esteem among sexual minority men (Vogel et al., 2014; Zervoulis et al., 2020). As they engage in self-presentation and social interaction on the apps, men encounter numerous discourses of stigma related to body, race/ethnicity, and gender expression.

Our interviewees narrated the experience of being the recipient or the perpetrator of stigma related to factors such as body, race/ethnicity, gender expression, and sexual position when communicating with other sexual minority men on apps. Such stigma has long occurred in physical community spaces such as bars (e.g., Loiacano, 1989), where sexual minority men have historically met, and continues to be experienced in such settings (e.g., Ruez, 2017). Stigma may manifest more overtly, however, on apps, where it may be enacted with more direct speech (e.g., an explicit statement about race preferences; Conner, 2019) compared with the indirect action (e.g., dismissing the advances of someone) that may occur in a space such as a bar.

The men in our study narrated compulsions to conform to particular body ideals consistent with other recent research (e.g., Chow, 2021; Conner, 2019; Tran et al., 2020). The experience of race/ethnicity stigma was central to the narratives of many men of color in our study, affirming recent research which has found proliferation of language of “preference” for White men or men of lighter skin tone on the apps (e.g., Conner, 2019; Tran et al., 2020). Finally, men’s narratives revealed pressures to conform to a particular performance of masculine gender presentation that denigrated femininity and thus reproduces misogyny among sexual minority men. This finding was consistent with emerging studies that highlight masculine gender performance on the apps (Cascalheira & Smith, 2020; Sarson, 2020) and its negative impact on men’s perceptions of their own masculinity (Miller & Brehm-Morawitz, 2020).

Counter to our expectations, narratives about stigma related to one’s HIV status, substance use, and social class did not emerge spontaneously. This finding may be related to the fact that the interviewees included in this analysis were young and socialized in a different era in relation to HIV (Hammack et al., 2018a). The availability of more effective treatment and prevention approaches has shifted the meaning of HIV in such a way that an HIV-positive status may be less stigmatizing (Hammack et al., 2019). Also, our interview protocol was not explicitly focused on such factors as substance use, HIV status, or social class, and interviewers did not probe for narratives related to these factors when they did not spontaneously occur within the interview.

Prior research focused on the experiences of community, sex, and HIV risk has demonstrated how these dimensions of stigma are intertwined for sexual minority men of color (e.g., Brennan et al, 2013; Han, 2006, 2008; Riggs, 2013). Similarly, our analysis revealed that when multiple forms of stigma were present, they were still deeply tied to racialized body and expression stereotypes (e.g., Black = hypermasculine, top, large penis, “thug”; Asian = slim, twink, effeminate; Latino = darker skin, “flamboyant”). Men of African, Latino, and Asian/Pacific Islander ancestry talked about how they experienced community in ways that positioned them against Whiteness, but White participants did not experience discreet body and gender expression stigma framed within racialized stereotypes.

Our findings demonstrate the importance of studying stigmatized identity traits among sexual minority men’s experiences in a racially and geographically diverse sample in ways that allow for the intersectional nature of stigma to emerge (Bowleg, 2013; Poon & Ho, 2008; Wilson et al., 2010). We urge those seeking to understand this population’s experiences to look beyond their silo (e.g., race, gender expression, body type) to the web of identities that inform these experiences. In an intersectional framework, sources of stress are not additive but rather mutually constitutive (Bowleg, 2013): men at the intersections of various identities appropriate or repudiate stigmatizing ideologies as they make meaning of their experience. Similar to the work of Black feminist theorists who identified the ways racist stereotypes for Black women have specific gender, body and personality attributes (Collins, 1986), our analyses suggest that an examination of multiple forms of stigma among racial and sexual minority men would be incomplete without considering the impact of racism in the production of stereotypes and expectations for body size, temperament, and sexual roles (Hutchinson, 1999).

Our intersectional approach has important implications for minority stress theory as it continues to develop. As a social theory of stress and health, conditions in the social environment related to stigma lie at its core (Meyer, 2003). The experience of stigma from within sexual minority communities highlights the way in which stress is experienced in nuanced ways linked to intersectional identities. The narratives of men in our study were impacted not only by structural disadvantage conferred by sexual minority identity in a heterosexist society but also by body, race/ethnicity, and gender expression in a society that privileges certain body types (e.g., thin, muscular), races (e.g., White), and gender expression (e.g., masculine). Our approach reveals the vitality of centering intersectionality as minority stress theory develops (see also Cyrus, 2017; McConnell et al., 2018; Ramirez & Galupo, 2019).

While our findings shed compelling light on intracommunity stigma in communities of sexual minority men, their limitations must be noted. First, our focus on men of a specific birth cohort of a specific age (i.e., 19–25 years old, born between 1990 and 1997) limits our ability to consider the ways in which older (e.g., Baby Boomers or members of Generation X) or younger (e.g., members of Generation Z) sexual minority men experience community and stigma. While men of this cohort came of age at a time when the apps represented key sites of connection for sexual minority men, men of older cohorts relied more on in-person settings for community engagement earlier in their development and thus may narrate their experience of the apps differently than the men in our study. Younger men (members of Generation Z) experienced key social movements such as MeToo and Black Lives Matter at earlier points in their development than men in our study, and their engagement on the apps is occurring at a time of greater awareness of stigma perpetuation on the apps. Thus, their experience may diverge from the men in our study.

Second, our qualitative sample provides descriptive and interpretive data based on a particular group of sexual minority men. The presence of even very compelling narratives does not address the question of how common such experiences are in the lives of sexual minority men in general. Third, since our interview protocol did not explicitly ask men about their experiences of stigma on the apps, we were limited to what emerged spontaneously in men’s narratives. The lack of reference to stigma based on substance use, HIV status, or social class does not suggest that men do not experience these forms of stigma. Finally, although our sample was racially and geographically diverse, it was limited to men in the USA, which offers a very specific cultural setting for gender, race, and sexuality.

Further research in this field is needed. First, a study that specifically focuses on young sexual minority men’s experiences of intracommunity stigma on apps could further explore the issues we raised. Our interview protocol was not focused on the experience of intracommunity stigma. Rather, we examined how these narratives emerged organically in response to questions about social identities, communities, and sexual culture. It was clear from our analysis that men’s identities and self-esteem were linked to the encounters they have on mobile apps and, therefore, understanding how they are experiencing stigma through this form of community engagement is vital. A study on how other generations of sexual minority men experience stigma on apps is also needed, as older men are likely to interact with technology in a different manner and may link their identities to these apps differently than younger men. Finally, understanding the broader impact that intracommunity stigma may have on community cohesion is necessary to provide further support for community-wide interventions; only through a robust understanding of this phenomenon informed by multiple methods can we recommend thoughtful and impactful campaigns with the goal of diminishing intracommunity stigma among sexual minority men.

An important consideration that these findings bring to the forefront is the role of community affiliation and participation for sexual minority men. As the source of community meaning has gradually shifted from in-person through such venues as bars and community centers to online through venues such as mobile apps, the historically protective role of the community to buffer minority stress may be diminished. The experience of community through apps appears to be more exclusively focused on seeking sex (Zervoulis et al., 2020), and the lack of face-to-face interaction may be associated with greater propensity for expressions of stigma such as racism (e.g., Conner, 2019; Jaspal, 2017; Tran et al., 2020).

While being a member of a sexual minority community may provide vital social support for some, it may also represent a challenge to others who do not experience feelings of belonging (Frost et al., 2016). As many have discussed, there is no one monolithic sexual minority community but rather an array of relatively discrete subgroups or communities; the members of these subgroups tend to feel a stronger affinity for their respective communities than for the overarching, more nebulous “LGBT community” (Holt, 2011; McCutcheon & Morrison, 2021; Peacock et al., 2001). Future research with sexual minority men might more closely interrogate their experience of community in smaller units and beyond the experience of mobile apps.

By highlighting the centrality of mobile apps in the experience of intracommunity stigma, this study provides insights into targeted clinical and public health (i.e., community-wide) interventions. Therapists, counselors, and public health interventionists can help to mitigate intracommunity stigma in sexual and gender minority communities. App designers might also work to create inclusive environments and educate their users about intracommunity stigma. For example, Grindr launched its “Kindr” campaign in 2018 to try to promote more inclusive and less explicitly stigmatizing discourse on its platform (Grindr, 2018). While the impact of this campaign on experiences of stigma remains to be investigated, research suggests that “polite incivility” remains on the platform following the campaign (Mowlabocus, 2021).

Expanding beyond apps, sexual and gender minority communities might initiate programs that work to create a more inclusive community for young sexual minority men. Such interventions might occur in many community outlets, such as community centers, but also where many young men are—online. For example, a coordinated media campaign intended to raise awareness of and ultimately reduce intracommunity stigma might occur directly on the apps, as well as on other social media sites such as Twitter and Instagram. Something akin to the media campaigns that have promoted PrEP and helped to destigmatize its use in HIV prevention seem worthy to explore (see Hammack et al., 2019).

### Conclusion

Young sexual minority men in diverse regions of the USA who experienced the emergence of mobile apps during early emerging adulthood spontaneously narrated the apps as a key source of community. Their narratives revealed the way in which cultural ideologies that privilege particular body types, races or ethnicities, and performances of masculine gender are encountered directly on mobile apps as men seek sex and connection. In this way, apps constitute contexts in which men must navigate stigma. The experience of stigma from other sexual minority people may compound the minority stress men already experience due to their sexual minority identity (Meyer et al., 2008; Pachankis et al., 2020).

Our study contributes to the growing literature on sexual minority men’s experience of mobile apps and the perpetuation of intracommunity stigma. The unique contribution of our study lies in its contextual grounding inherent in a life course theoretical approach, which highlights the social location of participants in history, and its intersectional framing, which allowed us to interrogate how forms of stigma (e.g., racism, misogyny) interact to create unique challenges as men navigate power and privilege.

Forms of intracommunity stigma experienced by the men in this study are not unique to sexual minority communities; the stigma narrated by men are akin to those present in the broader American society. Forms of stigma these men narrated are consistent with what tend to be viewed as the idealized traits of a heterosexual man in our heteronormative society: racially White, physically powerful, gender conforming, and sexually dominant (e.g., Abelson, 2019; Callen, 2018; Han & Choi, 2018). The reproduction of this heteronormative ideal in men’s personal narratives may reflect internalized heterosexism within communities of sexual minority men, thus reflecting the problematic allure of a particular masculine gender ideology which perpetuates various forms of stigma.

## Data Availability

Qualitative data reported in this article are not publicly available per informed consent agreement with interviewees and procedures approved by the UCLA IRB.
